# Macrophage phenotypic subtypes diametrically regulate epithelial-mesenchymal plasticity in breast cancer cells

**DOI:** 10.1186/s12885-016-2411-1

**Published:** 2016-07-07

**Authors:** Min Yang, Bo Ma, Hanshuang Shao, Amanda M. Clark, Alan Wells

**Affiliations:** Department of Pathology, University of Pittsburgh, and Pittsburgh VA Health System, Pittsburgh, PA USA; Current address: Institute of Materia Medica, Chinese Academy Medical of Sciences & Peking Union Medical College, 1 Xian Nong Tan Street, 100050 Beijing, China

**Keywords:** Macrophage polarity, Mesenchymal to epithelial reverting transition, Epithelial to mesenchymal transition, Metastatic microenvironment

## Abstract

**Background:**

Metastatic progression of breast cancer involves phenotypic plasticity of the carcinoma cells moving between epithelial and mesenchymal behaviors. During metastatic seeding and dormancy, even highly aggressive carcinoma cells take on an E-cadherin-positive epithelial phenotype that is absent from the emergent, lethal metastatic outgrowths. These phenotypes are linked to the metastatic microenvironment, though the specific cells and induction signals are still to be deciphered. Recent evidence suggests that macrophages impact tumor progression, and may alter the balance between cancer cell EMT and MErT in the metastatic microenvironment.

**Methods:**

Here we explore the role of M1/M2 macrophages in epithelial-mesenchymal plasticity of breast cancer cells by coculturing epithelial and mesenchymal cells lines with macrophages.

**Results:**

We found that after polarizing the THP-1 human monocyte cell line, the M1 and M2-types were stable and maintained when co-cultured with breast cancer cells. Surprisingly, M2 macrophages may conferred a growth advantage to the epithelial MCF-7 cells, with these cells being driven to a partial mesenchymal phenotypic as indicated by spindle morphology. Notably, E-cadherin protein expression is significantly decreased in MCF-7 cells co-cultured with M2 macrophages. M0 and M1 macrophages had no effect on the MCF-7 epithelial phenotype. However, the M1 macrophages impacted the highly aggressive mesenchymal-like MDA-MB-231 breast cancer cells to take on a quiescent, epithelial phenotype with re-expression of E-cadherin. The M2 macrophages if anything exacerbated the mesenchymal phenotype of the MDA-MB-231 cells.

**Conclusion:**

Our findings demonstrate M2 macrophages might impart outgrowth and M1 macrophages may contribute to dormancy behaviors in metastatic breast cancer cells. Thus EMT and MErT are regulated by selected macrophage phenotype in the liver metastatic microenvironment. These results indicate macrophage could be a potential therapeutic target for limiting death due to malignant metastases in breast cancer.

**Electronic supplementary material:**

The online version of this article (doi:10.1186/s12885-016-2411-1) contains supplementary material, which is available to authorized users.

## Background

Recurrences at metastatic sites represent a major cause of mortality in breast cancer patients [1, 2]. It is reported that 20–45 % of breast cancer patients will relapse years or even decades after the resection of the primary tumor [3]. Only a small number of the disseminated tumor cells that lodge in secondary organs will eventually grow to form a clinically evident metastasis; cancer cells can remain dormant in secondary organs for years [4, 5]. The existence of such dormant cancer cells at metastatic sites has been described previously as quiescent solitary cells that neither proliferate nor undergo apoptosis [1, 6]. Therefore, a comprehensive understanding of the “switch” from a dormant to a growth state is crucial to elucidate the mechanism of cancer progression and recurrence, might lead to the development of novel treatments for cancer metastasis.

The cancer-associated Epithelial-to-Mesenchymal Transition (EMT) has been strongly correlated with metastasis and shortened life expectancy of many carcinomas, has been proposed as a mechanism for enabling cancer cell invasion and dissemination [7, 8]. Nevertheless, EMT is reversible and that a reversion back towards the epithelial phenotype may occur at the secondary metastatic site (MErT) [9]. Current opinion and our previous studies revealed that metastatic breast cancer dormancy is likely not sustainable by the invasive, mesenchymal phenotype but rather through a partial epithelial reversion in which the cells are in a quiescent state [9, 10]. Accumulating evidence suggests that MErT may be critical for breast cancer ectopic survival and dormancy once a distant metastasis is involved. Furthermore, studies have shown that a secondary epithelial to mesenchymal transition is thought to underlie latent metastatic outgrowth [10–12]. Therefore, EMT and MErT may determine dormant or active states of the tumour, respectively, and allow for an indeterminate number of metastases formation.

It is established that distant metastases involves disseminated tumor cells adapting to the foreign environment, suggesting that the microenvironment is capable of regulating a series of switches between EMT and MErT phenotypes [13–15]. The triggers for the second mesenchymal transition of these dormant cells are not known though initial studies in an ex vivo microphysiological system suggest that inflammatory signals may underlie this [16, 17]. In breast cancer stroma, key cells of the innate inflammatory process, macrophages, can occupy more than 50 % of the breast tumour mass and influence breast cancer prognosis [18, 19]. Macrophages are heterogeneous in population and can be classified within a spectrum of M1 or M2, polarising to each dependent on the stimuli present at time of activation. Recently, it has been shown that tumor-associated macrophages (TAMs), which are characterized by M2 macrophages, contribute to EMT and cancer metastasis from primary tumor to a distant tissue [20, 21]. However, the roles of macrophages on modulating the balance between EMT and MErT of breast cancer cells in response to a metastatic microenvironment remain unclear.

The goal of this study was to determine the functional contributions of M1/M2 macrophages to epithelial-mesenchymal plasticity in breast cancer cells, and to elucidate the underlying effects of macrophage polarization on tumor dormancy or growth state for emergency in metastatic tumors. This work might highlight a novel function of macrophage for colonization in the distant metastatic site, and serve as a foundation to explore mechanisms to either maintain metastatic dormancy or induce emergence.

## Methods

### Antibodies and reagents

Phorbol 12-myristate 13-acetate (PMA), Lipopolysaccharides (LPS) and Hoechst 33258 dye were from Sigma-Aldrich (St. Louis, MO, USA). The cytokines for interferon γ (IFNγ), interleukin-4 (IL-4) and interleukin-13 (IL-13) were from R&D Systems (Minneapolis, MN, USA). The antibody for CD163 was from Abcam (Cambridge, MA, USA). The primary antibodies for E-cadherin, GAPDH were from Cell Signaling Technology (Danvers, MA, USA). Antibodies for fluorescein isothiocyanate (FITC)-conjugated CD68, FITC-conjugated CD206, FITC-conjugated CD163, FITC-conjugated HLA-DR and isotype control were from BD Biosciences (San Jose, CA, USA). FITC-conjugated E-cadherin antibody was from BioLegend (San Diego, CA, USA). Hepatocyte maintenance medium (HMM) and SingleQuots were from Lonza (Anaheim, CA, USA).

### Cell culture and differentiation

The breast cancer cell lines MCF7, MDA-MB-231 and the human THP-1 cell line were purchased from ATCC (Manassas, VA, USA). MDA-MB-231 and MCF7 cell lines were transfected with red fluorescent protein (RFP) as previously described [9]. To maintain selection for RFP positive breast cancer cells, MCF-7 cells were cultured with 900 μg/ml G418, and MDA-MB-231 were cultured with 5 μg/ml puromycin in RPMI-1640 (Life Technologies, Carslbad, CA) supplemented with 10 % FBS until used in the experiments. THP-1 cells were cultured in RPMI 1640 supplemented with 10 % FBS, 100 mg/ml penicillin, and 100 mg/ml streptomycin (Gibco, Life Technologies, Grand Island, NY, USA).

THP-1 cells were differentiated and polarized according to established protocols [20, 27], with minor modifications. Briefly, THP-1 cells were plated in RPMI media and treated with 320 nM PMA for 72 h, cells that adopted an adherent macrophage-like phenotype were selected as M0 macrophage. To generate M1-polarized macrophages, THP-1 cells were treated with 320 nM PMA for 6 h and then cultured with PMA plus 100 ng/ml LPS and 20 ng/ml IFNγ for the remaining 66 h of a total incubation time of 72 h. To generate M2-polarized macrophages, THP-1 cells were treated with 320 nM PMA for 6 h, and then cultured with PMA plus 20 ng/ml IL-4 and 20 ng/ml IL-13 for another 66 h of a total incubation time of 72 h.

Peripheral blood mononuclear cells (PBMCs) were obtained using a previously described method [22, 23]. Briefly, we mixed the human peripheral blood sample with an equal volume of room temperature PBS, pelleted the leukocyte/RBC fraction by centrifuging the cells 15 min at 200 × *g*, at room temperature. The supernatant was removed and the leukocyte/RBC cell suspension brought to a final volume of 40 ml with 1× PBS. The 10 ml leukocyte/RBC/PBS mixture was layered over 3 ml Ficoll-Hypaque (10771 SIGMA) solution and centrifuged 20 to 30 min 900 g, 18° to 20 °C, with no brake. The mononuclear lymphocyte cell were washed by adding HBSS (∼3 times the volume of the mononuclear cell layer) and centrifuging 10 min at 450 to 600 × *g*, 18° to 20 °C. Mononuclear cells were resuspended in RPMI-1640 medium and seeded on poly-L-Lysine coated coverslips. Media was changed after 4 h to remove un-attached cells. For polarization we added 50 ng/ml GM-CSF for M1 or 50 ng/ml M-CSF for M2, and replenished after 48 h for total 96 h. Media was then supplemented with LPS (100 ng/ml) and IFNγ (20 ng/ml) for M1 or IL-4 (20 ng/ml) and IL-13 (20 ng/ml) for M2 for 72 h. We seeded 200,000 RFP-labeled MCF-7 or MDA-MB-231 onto the macrophages monolayer in HMM medium after thoroughly wash to remove cytokines. Cells were fixed after 5 days.

### Macrophage and breast cancer cell co-culture

One million THP-1 cells, or primary human monocyte-derived macrophages, were plated in 6-well plates in 2-mL RPMI1640 with 10 % FBS plus PMA and M1-trophic (IFN-γ and LPS) or M2-trophic (IL-4 and IL-13) cytokines treatment for 72 h to induce differentiation into M1/M2 macrophages. M0 macrophages, cells treated with only PMA were taken as controls. After a thorough wash to remove PMA and cytokines, 2 × 10^5^ per well MCF7 or MDA-MB-231 cells were seeded onto the M1/M2, M0 macrophages monolayer and co-cultured in HMM medium for additional 5 days. Breast cancer cell lines were alone planted in HMM medium as the control group.

In addition, the M0, M1 and M2- conditioned media (CM) was collected everyday for breast cancer cells treatment. The culture supernatants from macrophages were collected 24 h later, by centrifuging at 500 × g for 5 min, as the macrophage CM. After 24 h, the normal growth medium RPMI1640 was discarded, MCF-7 or MDA-231 cells were treatment M0, M1 or M2 CM was added totally for 5 days, and changed M0, M1 or M2 CM everyday.

Co-culture systems were established by using transwell inserts (0.4 mm pore, polycarbonate membrane; Costar, Cambridge, MA, USA) and transferred to six-well culture plates. M0, M1 or M2 macrophages (1 × 10^6^ cells) were loaded in the upper inserts, and MCF7 or MDA-MB-231 cells (2 × 10^5^ cells) were put into the lower compartment of the culture well and serum-starved for at least 12 h before co-culture. Macrophages co-cultures with MCF-7 or MDA-MB-231 cells were performed in serum free HMM for 5 days. Total cellular protein was extracted from MCF7 or MDA-MB-231 cells exposed to different experimental conditions.

### Flow cytometry

Phenotypic changes in macrophages were verified by flow cytometry using the BD LSRFortessa flow cytometer (BD Biosciences, Franklin Lakes, NJ). After blocking human FcRs, cells were washed and resuspended in PBS supplemented with 1 % heat-inactivated FBS and 0.01 % NaN3. For CD68 staining, cells were fixed and permeabilized with a BD Cytofix/Cytoperm Fixation/Permeabilization Solution Kit (BD Biosciences, Franklin Lakes, NJ). Cells were then incubated with the FITC-CD68 antibody. For surface markers, cells were incubated with FITC-coupled antibodies to CD206, CD163, HLA-DR, as well as isotype matched control antibodies, for 30 min at 4 °C in the dark. Flow cytometry analyses of co-cultures were accomplished by non-enzymatic dissociation of cells from the culture plates. The cells were fixed in 2 % Paraformaldehyde for 30 min, permeabilized with 1 % Triton for 3 min, and incubated with a FITC-conjugated E-cadherin antibody for 30 min. Following the final washing step, labeled cells were analyzed by flow cytometry using BD FACSDiva Software (BD Biosciences, Franklin Lakes, NJ). The mixed macrophage-breast cancer cell suspension was gated as to identify breast cancer cells using RFP fluorescence versus side scatter. Gates were set on the RFP+ population, and this gated population was analyzed for E-Cadherin expression. The RFP- population was analyzed for macrophage marker expression.

### Imaging

Phase contrast images of whole cell morphology were captured by an Olympus inverted scope and digitally analyzed using Spot Advanced software (Diagnostics Instruments, Macomb, MI). Immunofluorescent staining of co-cultures was conducted to evaluate E-cadherin expression. Cell slides were incubated with the primary antibody for E-cadherin (1: 500) at 4 °C overnight and then with Alexa Fluo® 488-conjugated secondary antibody at room temperature for 1 h. Counterstains used were 0.1 % Hoechst 33258 dye for nuclei. Immunofluorescent images were captured on an Olympus Fluoview 1000 scope (Olympus, Center Valley, PA) and captured using Fluoview Viewer.

Images from co-cultures in HMM versus MCF-7 or MDA-MB-231 in RPMI growth medium were imported into ImageJ Version 1.44i (U. S. National Institutes of Health, Bethesda, Maryland). Two fields per experimental condition were analyzed. Cell perimeters and midpoint widths were manually traced and measured in pixel units using the ImageJ functions. The ratio of width versus perimeter was computed for each cell and the mean values, standard deviations, and Student’s t test (2 tailed) were calculated using Prism 5 (GraphPad Software, San Diego, CA, USA).

Breast cancer cells viability was assessed as the quantity of RFP+ cells to the total cell number of co-cultures. Manual calculations of the RFP+ cells within 10 random fields with approximately 500–1000 cells were performed.

### Xenografts

Metastatic samples in mice: Prostate cancer (PCa) DU145 cells were obtained from ATCC, cultured in RPMI-1640 media supplemented with 10 % fetal bovine serum (FBS) in the presence of 100 u/mL penicillin and 0.1 mg/mL streptomycin. Seven-week-old male NOD/SCID gamma mice were purchased from the Jackson Laboratories (Bar Harbor, ME). For the in vivo hepatic metastasis model cells were injected into the spleen and both liver and spleen examined. After anesthetizing the mice, a transverse incision in the left flank was made to expose the spleen; a half a million viable PCa DU145 cells were injected into the spleen using a 27-gauge needle. Five weeks after inoculation, the tumor xenograft tissues of NOD/SCID gamma mice were fixed in 10 % neutral buffered formalin for immunohistochemical staining. The Mouse on Mouse Kit (Vector Labs, Berlingame, CA), was used for positive labeling by comparing serial sections incubated with the primary antibodies for E-cadherin or CD163, and the biotinylated secondary antibody alone. Labeling was visualized with the Vectastain Elite kit (Vector Labs). The AAALAC-accredited Institutional Animal Care and Use Committees of the Veteran’s Administration Pittsburgh Health System approved all animal studies and procedures.

### Western blot

Cells were lysed in SDS buffer (20 mM Tris-HCl pH 7.4, 150 mM NaCl, 5 mM EDTA, 5 mM EGTA and 1 % SDS) containing complete protease inhibitor cocktail (Roche). Protein concentration was determined by the Bradford protein assay and gels loaded with equal amounts of protein per lane. A 8 % SDS–PAGE gel resolved cell lysates and were subsequently transferred to a PVDF membrane. Membranes were blocked with 5 % serum albumin for 1 h and incubated overnight with primary antibody E-cadherin or GAPDH. The membranes were washed and incubated with peroxidase-conjugated secondary antibodies for 1 h, and then detected by enhanced chemiluminescence.

### Statistical analysis

Data analysis involved use of GraphPad software (GraphPad Prism version 5.00 for Windows, GraphPad Software, San Diego, CA, USA). Results are expressed as mean ± s.e.m. Differences were analyzed by t-test or ANOVA, and results were considered significant at *P <*0.05.

## Results

### M1 or M2 phenotype is stable following co-culture with breast cancer cells in hepatic microphysiologic system

**Fig 1:**
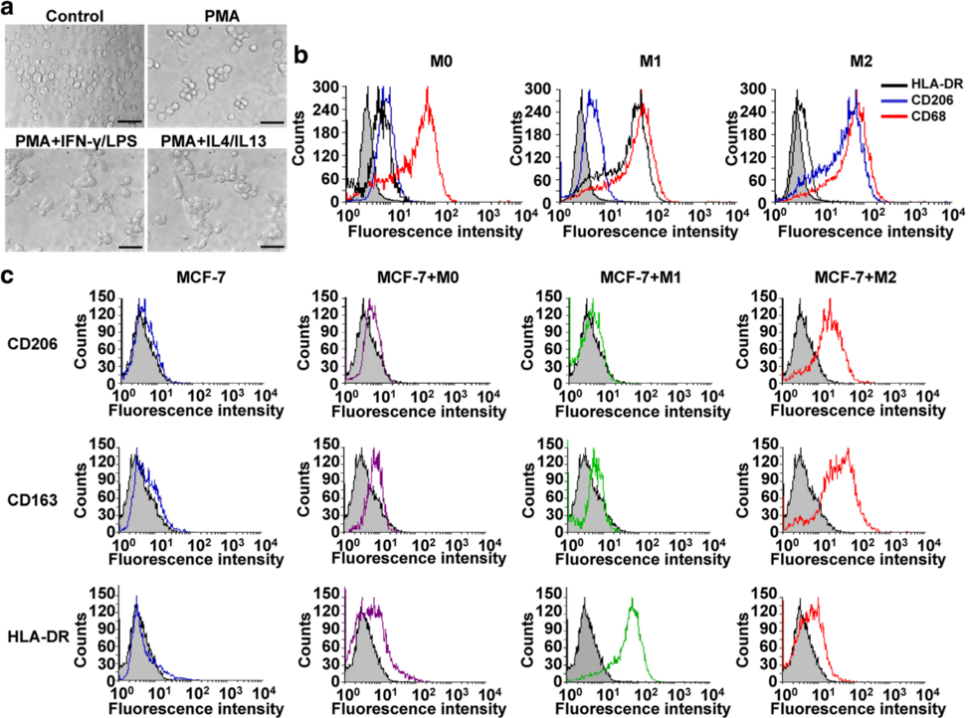
Characterization of THP1-derived M1- or M2-like macrophages when co-cultured with breast cancer cells for 5 days. **a** THP-1 cells were PMA-treated for 6 h, with addition of M1-polarizing or M2-polarizing cytokines during the final 66 h of treatment. Morphological changes in response to M1-trophic or M2-trophic cytokines stimuli. (Bar = 50 μm). **b** Flow cytometry based analysis of M0, M1 and M2 macrophages for HLA-DR, CD206 or CD68 expression. The grey background represents unstained control. **c** Flow cytometric analysis of the indicated markers on M0, M1 and M2 macrophage subsets by breast cancer cells co-culture. M0, M1 or M2 macrophages co-cultured with MCF-7 cells in serum-free hepatocyte maintenance medium (HMM) for 5 days, mono-cultured MCF-7 in their normal growth medium (RPMI 1640 with 10 % serum). CD206 and CD163 are both markers for M2 macrophages. HLA-DR is the marker for M1 macrophages. The *blue line* indicates mono-cultured MCF-7 cells, the *purple line* indicates M0 macrophages co-cultured with MCF-7 cells, the *green line* indicates M1 macrophages co-cultured with MCF-7 cells, the *red line* indicates M2 macrophages co-cultured with MCF-7 cells. Shown are represented of at least three independent experiments

Human THP-1 cells are a widely used model for differentiated tissue macrophages that closely resembles native monocyte-derived macrophage differentiation [24]. To explore the interaction between macrophages and breast cancer cells, we used M1- or M2-polarized THP-1 macrophages in co-culture with breast cancer cells. As the ultimate goal is to determine the macrophage role in metastatic breast cancer cell dormancy we chose a liver tissue medium that allows for breast cancer survival, but is devoid of serum, HMM medium. The protocol for the experiment is shown in Additional file 1: Figure S1, THP-1 cells were treated with PMA for 72 h after which fully differentiated macrophages were generated (as ‘M0’ in Fig. 1). At this stage, THP-1 cells were treated with PMA for 6 h, followed by stimulation of either IFNγ/LPS or IL-4/IL-13 for a resting period of 72 h to obtain M1- and M2-like phenotypes, respectively (denoted as ‘M1’ and ‘M2’, Fig. 1). Morphology analysis showed that undifferentiated THP-1 cells were loosely suspended round cells. When treated with PMA, THP-1 cells quickly stopped proliferating, became attached, differentiated into tightly adherent (Fig.1a). Interestingly, M1/M2-polarized THP-1 macrophages displayed a fully differentiated amoeboid morphology including oval, elongated, or pseudopodia-like cells compared with the spherical shape of M0 macrophages (Fig.1a). These morphological changes of the macrophages were similar to those described earlier [12].

Phenotypes were confirmed via flow cytometric analysis of standardized cell surface markers. As shown in Fig.1b and Additional file 2: Figure S2a, the expression of macrophage marker CD68 was markedly upregulated on PMA-treated macrophages, indicating that THP-1 monocytes were well differentiated to macrophages. M1 macrophage marker (HLA-DR) was markedly increased with IFNγ/LPS pre-treatment, while the expression of HLA-DR was very low with IL-4/IL-13 pre-treatment. Moreover, results showed that the level of M2 macrophage–specific marker CD206 was upregulated in IL-4 plus IL-13 treatment, whereas IFNγ/LPS did not affect CD206 expression (Fig.1b and Additional file 2: Figure S2a).

To further validate that this protocol was efficient in eliciting M1 and M2-like polarization in the THP-1 cells that was stable for the experimental time course. Following 5 days of co-culture with breast cancer cells in HMM medium, we found that the M1 or M2 macrophage markers in co-cultured cells. The mixed macrophage-breast cancer cell suspension was gated as to exclude breast cancer cells using RFP fluorescence versus side scatter. Gates were set on the RFP- population, and this gated population was analyzed for M1 or M2 macrophages. Flow cytometric analysis revealed the levels of M2 macrophage marker (CD206, CD163) and M1 macrophage marker (HLA-DR) were very low in MCF-7 cells culture alone, suggested no expression of macrophage marker in breast cancer cells (Fig.1c and Additional file 2: Figure S2b). After 5 days of co-culture cells, we found that the IL-4/IL-13 pre-treatment led to significantly increased expression of M2 macrophage–specific markers CD206 and CD163, whereas IFNγ/LPS pre-treatment resulted in upregulated level of M1 macrophage–specific marker HLA-DR with no effect on the expression of upregulated CD206 and CD163 (Fig.1c and Additional file 2: Figure S2b). Together, these data indicated that pre-polarized M1 and M2 macrophages retained their phenotype even after coculturing with MCF-7 cells.

### M1 and M2 macrophages differentially affect breast cancer cells outgrowth in HMM medium

To investigate the effects of M1 and M2 macrophages on breast cancer cells numbers, we observed the outgrowth of RFP positive MCF-7 cells and RFP positive MDA-MB-231 cells within the co-culture system in HMM medium via imaging. The noninvasive breast cancer line MCF-7 cells and highly invasive and metastatic MDA-MB-231 cells exogenously expressing RFP were seeded under three culture conditions: (1) co-cultured with M0, M1 or M2 macrophages in serum-free HMM, (2) mono-cultured RFP-MCF-7 or RFP-MDA-MB-231 in serum-free HMM as the negative control, and (3) mono-cultured RFP-MCF-7 or RFP-MDA-MB-231MCF-7 in their normal growth medium (RPMI with 10 % serum) as the positive control.

**Fig 2:**
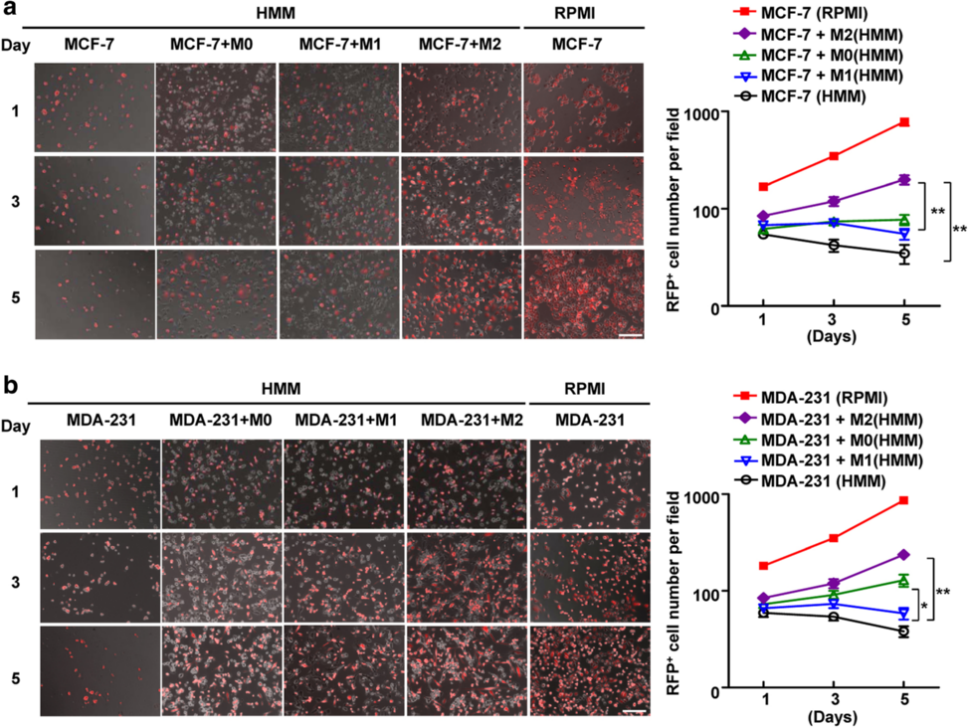
Changes in cell viability of breast cancer cells following co-culture with M1 or M2 macrophages. **a** M2 macrophages may confer a growth advantage to MCF-7 cells. A Phase contrast imaging with a fluorescence overlay (Red MCF-7 cells) are representative images on Day 1, Day 3 and Day 5 across 5 conditions. (Bar = 100 μm). MCF-7 cells co-cultured with M0, M1 or M2 macrophages in serum-free hepatocyte maintenance medium (HMM), MCF-7 cells mono-cultured in serum-free HMM as the negative control, mono-cultured MCF-7 in their normal growth medium (RPMI 1640 with 10 % serum) as the positive control. **, *P* <0.01. **b** M1 macrophages may inhibit a growth advantage of MDA-MB-231 cells by microscope on days 5. A Phase contrast imaging with a fluorescence overlay (Red MDA-MB-231 cells) are representative images on Day 1, Day 3 and Day 5 across 5 conditions. (Bar = 100 μm). *, *P* <0.05. **, *P* <0.01. For both (**a**) and (**b**), images are representative of three independent experiments each in triplicate

Measurement of RFP positive MCF-7 cells by microscopy revealed a slowly decreasing number of MCF-7 cells in HMM medium was observed throughout the 5-day experimental timeframe (negative control), while the MCF-7 cells significantly increased in RPMI 1640 with 10 % serum (positive control) (Fig.2a). At day 3, MCF-7 cells co-cultured with M0 or M1 macrophages in serum-free HMM medium maintain low survival and outgrowth of MCF-7 cells compared with MCF-7 cells mono-cultured in serum-free HMM, there was no statistically significant difference in cell viability of MCF-7 cells among these groups (*P* >0.05, ANOVA). Moreover, there was also no significant difference in MCF-7 cells outgrowth in co-culture with M0 or M1 macrophages (Fig. 2a). Notably, the MCF-7 cells substantially increased after 5 days of co-culture with M2 macrophages in HMM as observed by microscopy (Fig. 2a), indicating that the M2 macrophages contribute to a growth advantage of MCF-7 cells.

We also found a slow decrease in RFP positive MDA-MB-231 cells in HMM medium for 5 days, but within the normal growth medium, MDA-MB-231 cells experienced exponential growth (Fig. 2b). Interestingly, the MDA-MB-231 cell number was substantially reduced after 5 days of co-culture with M1 macrophages in comparison with M2 or M0 co-culture (Fig. 2b). These results demonstrated M2 macrophages may confer a growth advantage to breast cancer cells.

### M2 macrophages promote a mesenchymal phenotype in MCF-7 cells

**Fig. 3:**
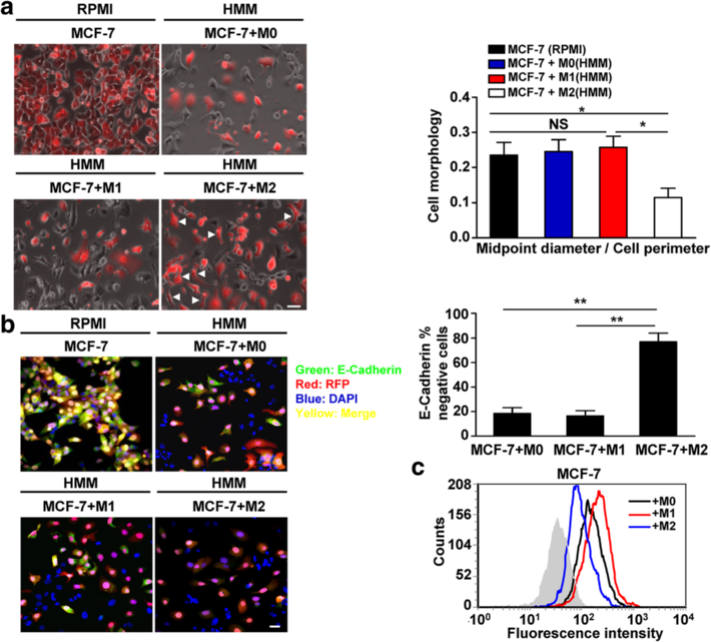
M2 macrophages confer a partial mesenchymal phenotypic shift of MCF-7. **a** Fluorescent imaging depicts RFP-MCF-7 cells co-cultured with M0, M1 or M2 macrophages in serum-free hepatocyte maintenance medium (HMM) for 5 days. MCF-7 cells mono-cultured in serum-free HMM on day 5 as the negative control, mono-cultured MCF-7 in their normal growth medium (RPMI 1640 with 10 % serum) on day 5 as the positive control. (Bar = 10 μm). *Arrows* indicate more elongated, spindle-shaped and mesenchymal-like cells. MCF-7 mesenchymal phenotypic shift in a quantified by the ratio of the midpoint diameter divided by cell perimeter. Data are mean ± s.e.m of 3 independent experiments. **, *P* <0.01. NS indicates not significant. **b** MCF7 cells co-cultured with M2 macrophages decreased E-cadherin expression. Immunofluorescence analysis of E-cadherin expression in RFP-MCF7 cells by co-culture with M0, M1 or M2 macrophages. E-cadherin (*green*), RFP (*red*), DAPI (*blue*), Merge (*yellow*) (Bar = 10 μm). Quantification of the percentage of E-cadherin negative cells in RFP-MCF-7 cells co-cultured with M0, M1 or M2 macrophages. **, *P* <0.01. 100 cells per field in 10 different fields of each group were quantified. Data are represented of at least three independent experiments. **c** Flow analysis of E-cadherin expression of RFP-MCF-7 population after 5 days of co-culture with M0, M1 or M2 macrophages. The *grey background* represents unstained control. The *black line* indicates co-culture with M0 macrophages. The *red line* indicates co-culture with M1 macrophages. The *blue line* indicates co-culture with M2 macrophages. Shown is one of two similar experiments

To explore the effect of macrophages on breast cancer cell phenotype, we co-cultured the epithelial MCF-7 cells with M0, M1 or M2 macrophages in HMM medium and observed RFP-MCF-7 cells morphology after 5 days of co-culture. As shown in Fig. 3a, MCF-7 cells exhibited their usual cobblestone appearance and tight cell-cell contacts in standard growth medium (1640 + 10%FBS). Importantly, in MCF-7 and M2 macrophages co-cultures, a sub-population of the MCF-7 cells transformed into more elongated, spindle-shaped cells, that did not readily form cell-cell contacts (Fig. 3a). For accurate evaluation of epithelial and mesenchymal morphologic phenotypes, we measuried the ratio of midpoint diameter to cell perimeter as described [12] using Image J software. Epithelial-shaped cells were cobblestone similar to cuboidal shape with a rounded aspect; the mesenchymal-shaped cells were elongated, spindle shape with low ratio of midpoint diameter to cell perimeter. This parameter reflects the morphological changes from epithelial cells to mesenchymal cells as occurs during EMT. MCF-7 cells polarity, ratio of midpoint diameter to cell perimeter, was significantly decreased by co-culture with M2 macrophages compared to that by co-culture with M1 or M0 macrophages (Fig.3a).

To assess the relationship between macrophages and breast cancer EMT, we detected the epithelial marker E-cadherin expression given that the loss of E-cadherin is a fundamental event in EMT. Immunofluorescence staining showed E-cadherin was substantially expressed in MCF-7 cells membrane that forms cell–cell adherins junctions in the RPMI controls (Fig.3b). E-cadherin expression was retained at the membrane of MCF-7 cells co-cultured with M0 or M1 macrophages in HMM medium (Fig.3b). However, E-cadherin protein expression was significantly decreased in MCF-7 cells co-cultured with M2 macrophages, and MCF-7 cells failed to maintain membrane E-cadherin expression (Fig.3b). In addition, there was no positive signal of E-cadherin expression for PMA-treated THP-1 cells. Flow cytometric analyses of MCF-7 cells after 5 days of co-culture with M2 macrophages displayed decreased E-cadherin expression in comparison to M1 or M0 macrophages co-culture (Fig.3c). To corroborate the findings in the primary cells, human monocyte-derived macrophages (MDMs) were utilized and E-cadherin staining was performed as mentioned above. Similar observation of E-cadherin expression was found in MDMs also (Additional file 3: Figure S3a). These data indicate that M2 macrophages could trigger EMT of MCF-7 cells in the liver metastatic microenvironment.

The in vivo relevance of this finding has been initially probed by examining the correlation between tumor cell phenotype and infiltrating macrophage polarization. We utilized a mouse model of prostate cancer DU145 cells inoculation into the spleen of NOD/SCID gamma mice with spontaneous dissemination to the liver. This model presents both E-cadherin-positive and –negative nodules as the tumor undergoes microenvironmentally-induced epithelial – mesenchymal plasticity. Immunohistochemical staining demonstrated that CD163, a marker of M2 macrophages, was rarely detected in the area where E-cadherin was prominently expressed but was markedly increased in hepatic metastatic tumors with low E-cadherin levels (Additional file 4: Figure S4). These initial, correlative data indicate that M2 macrophages infiltration significantly correlates with EMT in metastatic tumors.

### M1 macrophages drive a mesenchymal to epithelial transition, while M2 macrophages confer a more mesenchymal phenotypic shift in MDA-MB-231 cells

**Fig. 4:**
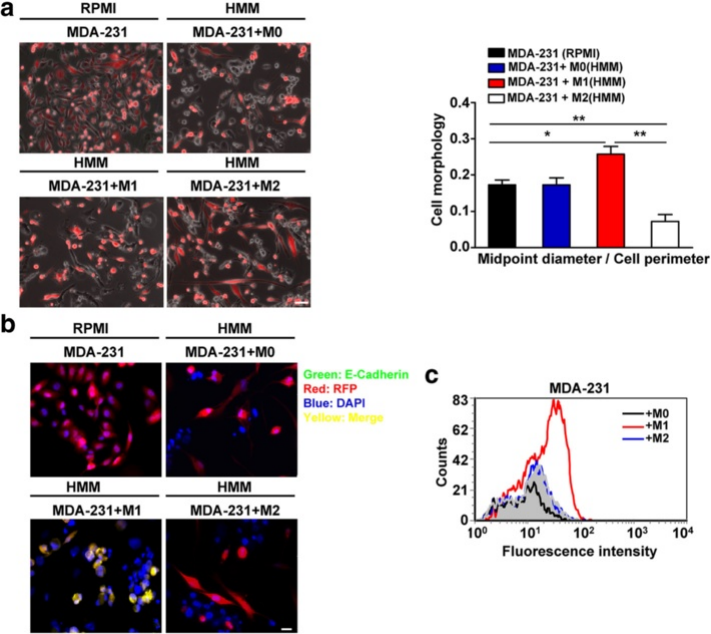
M1 macrophages accelerate mesenchymal to epithelial transition in MDA-MB-231. **a** Fluorescent imaging depicts MDA-MB-231 cells co-cultured with M0, M1 or M2 macrophages in serum-free hepatocyte maintenance medium (HMM) for 5 days. MDA-MB-231 cells mono-cultured in serum-free HMM on day 5 as the negative control, mono-cultured MDA-MB-231 in their normal growth medium (RPMI 1640 with 10 % serum) on day 5 as the positive control. (Bar = 10 μm). MDA-MB-231 phenotypic shift in a quantified by the ratio of the midpoint diameter divided by cell perimeter. Data are mean ± s.e.m of 3 independent experiments. *, *P* <0.05. **, *P* <0.01. **b** M1 macrophages drive the re-expression of E-cadherin in MDA-MB-231 breast cancer cells. Immunofluorescence analysis of E-cadherin expression in RFP-MDA-MB-231 cells by co-culture with M0, M1 or M2 macrophages. E-cadherin(*green*), RFP (*red*), DAPI (*blue*), Merge(*yellow*). (Bar = 10 μm). **c** Flow analysis of E-cadherin expression of RFP-MDA-MB-231 population after 5 days of co-culture with M0, M1 or M2 macrophages. The *grey background* represents unstained control. The *black line* indicates co-culture with M0 macrophages. The *red line* indicates co-culture with M1 macrophages. The *blue line* indicates co-culture with M2 macrophages. Shown is one of two similar experiments

Our previous study reported that hepatocytes drive MErT, characterized by the re-expression of E-cadherin in MDA-MB-231 cells [25]. To evaluate whether macrophages could induce MErT in breast cancer cells at the metastatic microenvironment, we co-cultured the mesenchymal-like MDA-MB-231 cells with M0, M1 or M2 macrophages in HMM medium and observed RFP-MDA-MB-231 cells morphology after 5 days of co-culture. As shown in Fig. 4a, MDA-MB-231 cells in standard growth medium (1640 + 10 % FBS) are characterized by a flattened, asymmetric morphology and do not form cell-cell contacts. Importantly, in MDA-MB-231 and M1 macrophages co-cultures in HMM medium, a sub-population of the MDA-MB-231 cells presented an epithelial shift as indicated by cobblestone morphology (Fig.4a). Interestingly, MDA-MB-231 cells became more elongated and spindle-shaped, and thus more mesenchymal after coculturing with M2 macrophages (Fig.4a). The analysis of cell polarity revealed that the ratio of midpoint diameter to cell perimeter in MDA-MB-231 cells was the highest by co-culture with M1 macrophages among co-culture cells or culture alone in normal growth medium, whereas which was significantly downregulated coculturing with M2 macrophages (Fig.4a).

To further confirm whether macrophages could induce an epithelial reversion as indicated by re-expression of E-cadherin, we assessed E-cadherin expression in the mesenchymal-like MDA-MB-231 cells after 5 days of co-culture in HMM medium by immunofluorescence. As shown in Fig.4b, E-cadherin was not expressed in MDA-MB-231 membrane in the RPMI controls. In addition, there was no positive signal of E-cadherin expression in MDA-MB-231 cells co-culture with M0 or M2 macrophages (Fig.4b). Notably, after 5 days of co-culture with M1 macrophages in HMM medium, E-cadherin was re-expressed in the sub-population of MDA-MB-231 cells that altered their cell shape (Fig.4b). Five (5) days of co-culture of MDA-231 cells with M1 macrophages increased expression of E-cadherin whereas this was not seen in M2 or M0 macrophage co-cultures as assessed by flow cytomteric analyses (Fig.4c). As with primary monocyte-derived macrophages, M1-polarized cells upregulated E-cadherin expression in MDA-MB-231 cells, while M0- or M2-polarized cells did not have any significant effect on E-cadherin expression (Additional file 3: Figure S3b).

To examine whether macrophages determine the balance between EMT and MErT in breast cancer cells via soluble factors, we used M1 and M2- conditioned media (CM) to see if they can affect E-cadherin expression in MDA-MB-231 or MCF7 cells. Western blot showed E-cadherin being highly expressed in MCF-7 cells, but there is no difference in E-cadherin expression with M0, M1 or M2 CM treatment compared to control groups (Additional file 5: Figure S5a). Similarly, M1 or M2 CM did not change the expression of E-cadherin in MDA-MB-231 cells compared with that in other groups (Additional file 5: Figure S5). Furthermore, co-cultures of M0, M1 or M2 macrophages and breast cancer cells in the transwell system showed the expression of E-cadherin did not differ in MCF7 or MDA-MB-231 cells (Additional file 5: Figure S5b). The findings suggest either that direct contact co-culture of breast cancer cells with macrophages is required to regulate epithelial-mesenchymal plasticity in the liver metastatic microenvironment or that the subset of cells that alter their phenotype is insufficiently large to affect this whole population assay. This latter possibility is unlikely given the relatively high percentage of MDA-MB-231 cells showing E-cadherin positivity with M1 co-culture or the fraction of MCF-7 cells demonstrating loss of E-cadherin in M2 co-culture.

## Discussion

In this study, we established in vitro models based on co-culture of breast cancer cells with macrophages in a liver microenvironment medium so as to investigate possible roles in regulating breast cancer metastasis dormancy and outgrowth. Using THP-1 cells (a human monocytic cell line) and a protocol we devised for their differentiation and polarization into M1- and M2-like populations, we found that dormancy and emergence from dormancy may hinge upon the M1 or M2 type of the macrophage polarization in the liver metastatic microenvironment, thus making the metastatic niche a potential therapeutic target to combat breast cancer metastasis.

The formation of micrometastases and the processes by which micrometastases progress to macrometastases are the main rate-limiting steps in clinically evident metastases [26]. Our foundational model posits that EMT and its accompanying reduction in E-cadherin expression enables carcinoma cells to disseminate from the primary tumor, while MErT with upregulation of E-cadherin expression allows disseminated carcinoma cells to integrate and survive within ectopic metastatic microenvironments [9, 12, 25, 27]. Outgrowth of these silent micrometastases is accompanied by a second EMT with subsequent formation of secondary macroscopic metastases [11, 28]. In addition to their role in constructing connections, the formation of cell heterotypic E-cadherin adhesions in the metastatic target organ, MErT may result in dormancy and enable the tumor cells to survive at a lower metabolic load at the micrometastasis stage [29, 30]. Thus the control of the epithelial-mesenchymal plasticity in breast cancer cells in secondary organs remains key to this mortal development. Therefore, we focused on the mechanism by which breast cancer cells regulate EMT/MErT balance in liver microenvironment.

Diverse interactions between the host microenvironment and cancer cells determine the course of tumor progression and metastasis. Macrophages are a major component of the breast cancer microenvironment. The human THP-1 cell line is a widely used monocyte/macrophage model, and the biologic behavior of THP-1 cells resembles monocyte-derived macrophages with respect to adherence, phagocytic capacity, and surface marker and cytokine expression [31, 32]. Therefore, we used the THP-1 monocyte cell line as a model for macrophage differentiation and polarization, and one with which to assess the effects of macrophages on epithelial-mesenchymal plasticity in breast cancer cells. The phenotypic state of the tumor cells is key to the fate of the micrometastases and also the chemosensitivity thereof [9, 25, 33].

Experimental and clinical data support the notion that the survivors persisting in metastatic sites can exist in at least three alternative states: (i) as solitary viable cancer cells in a quiescent, nonproliferative state (dormancy); (ii) as micrometastases, which remain as small, at least quasi-quiescent lesions; or (iii) as actively growing macrometastatic lesions [34]. Thus, the critical question as to whether the micrometastatic cells are in a state of quiescence or outgrowth is the key to dealing with tumor dormancy, and should be at the forefront of new approaches to tumor management. We previously reported that perturbations of the parenchymal hepatocytes and NPCs in the liver metastatic microenvironment may differentially contribute to metastatic dormancy, stability, or emergence [12].

In examining NPC subsets, tissue macrophages, including Kupffer cells, stand out. Macrophages represent one of the main stromal cell populations, but are diverse as they take on various functional phenotypes, referred to as M1 and M2 phenotypes. The role of macrophages in tumor progression has been shown to be double-edged. Pro-inflammatory, or classically activated M1 macrophages, exert resistance against tumors and elicit tissue disruptive reactions, while anti-inflammatory, or alternatively activated M2 macrophages, appear to have important roles in promoting tumor growth and metastasis [35–39]. But these earlier works all looked at the primary cancer situation. The actions in the metastatic site may be different if not diametric. Our study demonstrates that the epithelial, non-metastatic human immortalized breast cancer cell line MCF-7 is exacerbated by M2 macrophage co-culture, whereas the highly invasive and mesenchymal MDA-MB-231 cell outgrowth is attenuated by M1 macrophage co-culture. This provided evidence that the M2 macrophages provided a favorable outgrowth environment, but M1 macrophages served as surrogate dormant for the breast cancer cells in this culture system.

The metastatic microenvironment is composed of a complex milieu of external cues arising from the tumor, stroma, and parenchymal cells. It has been reported that stromal cells regulate proliferation and motility of cancer cells through both soluble factors and direct cell-cell interactions [40]. In the present study, it was suggested that cell-cell contact was required for macrophage phenotypic subtypes to regulate epithelial-mesenchymal plasticity in breast cancer cells. Recent reports have shown that direct cell-cell contact between M2 macrophages and cancer cells induced migration and invasion of cancer cells while M1 macrophages reduced cancer cell invasion [41]. However, the numerous cell surface and extracellular matrix (ECM) molecules potentially involved in cell-cell interaction are not identified yet, nor have the molecular mechanisms by which these changes actively induce or repress gene expression in malignant cells. In fact, two-dimensional (2D) culture did not recapitulate many of the complex properties of the three-dimensional (3D) in vivo microenvironment. One main issue is the stiffness of the supporting substratum. Pathological, and in tissue culture supra-physiological stiffness is well known to lead to a mesenchymal transition of even non-neoplastic breast epithelial cells [42, 43]. Our model system for the hepatic niche moves beyond organotypic modeling into microphysiologic systems, providing the mechanical stresses and oxygenation that is so important to the liver [17, 44]. As a part of our research we are continuing to explore the behaviors of cancer cells in the metastatic microenvironment to lie dormant or aggressively grow out through connection of this microphysiologic system with immune system activation.

There are several possible outcomes after cancer cells extravasate into a metastatic target tissue: apoptosis, dormancy, or sustained proliferation [45]. Epithelial-mesenchymal plasticity shifts may influence the discontinuous behavior of cancers, in which some cancers remain dormant for years after therapy, or to relapse and wreak havoc [46]. The formation of cell heterotypic E-cadherin adhesions in the metastatic target organ may result in dormancy at the micrometastasis stage. After this quiescence, a secondary insult, independent of carcinoma per se, to the local environment may induces renewed carcinoma cell proliferation and escape from E-cadherin-mediated contact inhibition during the metastatic seeding of disseminated carcinomas [29]. In this study, we report that macrophage phenotypic subtypes diametrically regulate epithelial-mesenchymal plasticity of breast cancer cells. Hence, epithelial-mesenchymal plasticity may be appropriate as a metastasis outgrowth prevention strategies in early stage carcinomas. Inducing the MErT program in dormant micrometastases by M1 macrophages would be a novel approach to maintain the long latency of a dormant stage, and prevent occurrence of late disease recurrence. Furthermore, since an increasing number of studies suggest a role of EMT in promoting chemoresistance [47], combining chemotherapies with M2 macrophages deletion holds promise to overcome chemoresistance in dormant tumor cells, thus providing a unique therapeutic approach to eradicate dormant tumor cells. In the near future, improving our understanding of the molecular regulation of the dynamic EMT/MErT programs during tumor metastasis will help to uncover new signaling pathways that can be therapeutically manipulated to either eliminate dormant tumor cells or to indefinitely maintain them in this dormant state, thus preventing a progressive metastatic disease.

## Conclusion

The current findings represent the first study to test the hypothesis and provide evidence that epithelial-mesenchymal plasticity in the metastatic cascade is regulated by selected M2 or M1 macrophages individually, which can serve as an efficient tool to explore the molecular mechanisms regulating metastatic tumor cell dormancy and the transition to metastatic growth. Our current study is limited by not fully examining the macrophage phenotypes in the complex micrometastatic niche; to achieve our ultimate goal of understanding cancer dormancy and progression in metastatic sites, we need to develop robust bioreactor models that incorporate appropriate tumor-host interactions. Still, this better understanding of the molecular regulation of the dynamic EMT/MErT programs during tumor metastasis will help to provide much-needed effective treatment to reducing the risk of recurrence.

## Abbreviations

CM, conditioned media; EMT, epithelial to mesenchymal transition; HMM, hepatocyte maintenance medium; IFN, interferon; IL, interleukin; LPS, lipopolysaccharides; M1, macrophage type 1; M2, macrophage type 2; MErT, mesenchymal to epithelial reverting transition; PMA, phorbol 12-myristate 13-acetate; TAMs, tumor-associated macrophages

## Additional files

Additional file 1: Figure S1. Schematic of experimental protocol to explore the role of macrophages on breast cancer cells outgrowth and epithelial-mesenchymal plasticity. Day 0, THP-1 cells were seeded in their normal growth medium (RPMI 1640 with 10 % serum). Day 3, Polarization of the monocytic THP-1 cell line into M1- or M2-like macrophages. Macrophages were co-cultured with RFP+ breast cancer cells (MCF-7 or MDA-MB-231), the medium was changed to serum-free maintenance HMM medium. Medium was changed every two days and breast cancer cells viability assayed routinely. Day 8, Identification of macrophage polarization and E-cadherin expression were examined by flow analysis or immunofluorescence staining. (JPG 526 kb) Additional file 2: Figure S2. Median fluorescence intensity (MFI) after staining with M1 or M2 macrophage markers and analysis by flow cytometry. The MFI served as a quantitative index of biomarker surface expression. **a** Quantitation of the MFI for CD68, HLA-DR, CD206 expression in M0, M1 and M2 macrophages. Data are mean ± s.e.m of three independent experiments. **, *P <*0.01. NS indicates not significant. **b** Quantitation of the MFI for CD206, CD163, HLA-DR expression in M0, M1 or M2 macrophages co-cultured with MCF-7 cells in serum-free hepatocyte maintenance medium (HMM) for 5 days, mono-cultured MCF-7 in their normal growth medium (RPMI 1640 with 10 % serum). Data are mean ± s.e.m of three independent experiments. **, *P <*0.01. (JPG 228 kb) Additional file 3: Figure S3. E-cadherin expression of breast cancer cell co-cultured with primary macrophage cell types. **a** Immunofluorescence analysis of E-cadherin expression in RFP-MCF7 cells after co-culture with M0, M1 and M2 programmed human monocyte derived macrophages (MDMs) in serum-free hepatocyte maintenance medium (HMM) for 5 days. E-cadherin (green), RFP (red), DAPI (blue), Merge (yellow) (Bar = 50 μm). **b** Immunofluorescence analysis of E-cadherin expression in RFP-MDA-MB-231 cells by co-culture with M0, M1 and M2 programmed human monocyte derived macrophages (MDMs) in serum-free hepatocyte maintenance medium (HMM) for 5 days. E-cadherin (green), RFP (red), DAPI (blue), Merge (yellow) (Bar = 50 μm). Shown are one of two similar experiments. (JPG 588 kb) Additional file 4: Figure S4. Immunohistochemical analysis of E-cadherin expression or M2 macrophage marker CD163 expression in metastatic hepatic foci of mouse model of prostate cancer dissemination. Metastatic foci in the liver were obtained from NOD/SCID gamma mice at 5 weeks after intrasplenic inoculation of prostate cancer (PCa) DU145 cells. The livers were fixed in 10 % neutral buffered formalin for immunohistochemical staining. Arrows indicate CD163 positive cells. (×200 magnification and scale bar = 50 μm). Shown are representative nodules of greater than five each. (JPG 1641 kb) Additional file 5: Figure S5. Direct contact co-culture of breast cancer cells with macrophages is required to regulate epithelial-mesenchymal plasticity. **a** MCF-7 or MDA-231 cells were co-cultured with M0, M1 or M2 conditioned media (CM) for 5 days. **b** MCF-7 or MDA-231 cells were co-cultured with M0, M1 or M2 macrophages in a transwell system for 5 days. MCF-7 or MDA-231 cells were also cultured in their normal growth medium (RPMI 1640 with 10 % serum) or in serum-free HMM. Western blot analysis of E-cadherin protein expression in MCF-7 or MDA-231 cells. GAPDH was used as a loading control. (JPG 390 kb)
