# Supplementary figures and images for: Macrophage phenotypic subtypes diametrically regulate epithelial-mesenchymal plasticity in breast cancer cells
Source: BMC Cancer. 2016 Jul 7;16:419. doi: 10.1186/s12885-016-2411-1 (PMC4936312; doi:10.1186/s12885-016-2411-1)

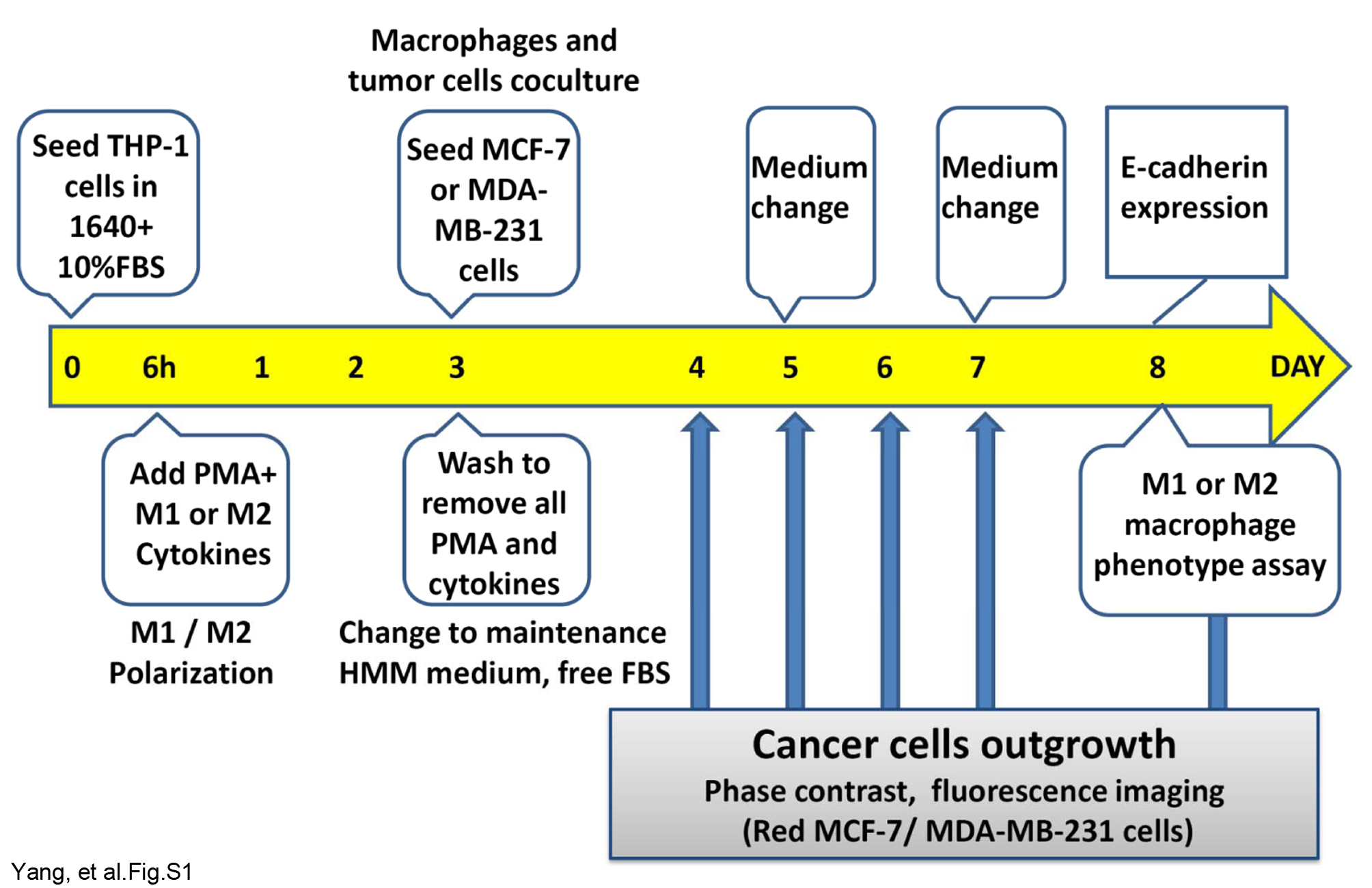

Supplement: Additional file 1: Figure S1. — Schematic of experimental protocol to explore the role of macrophages on breast cancer cells outgrowth and epithelial-mesenchymal plasticity. Day 0, THP-1 cells were seeded in their normal growth medium (RPMI 1640 with 10 % serum). Day 3, Polarization of the monocytic THP-1 cell line into M1- or M2-like macrophages. Macrophages were co-cultured with RFP+ breast cancer cells (MCF-7 or MDA-MB-231), the medium was changed to serum-free maintenance HMM medium. Medium was changed every two days and breast cancer cells viability assayed routinely. Day 8, Identification of macrophage polarization and E-cadherin expression were examined by flow analysis or immunofluorescence staining. (JPG 526 kb) [file 12885_2016_2411_MOESM1_ESM.jpg]

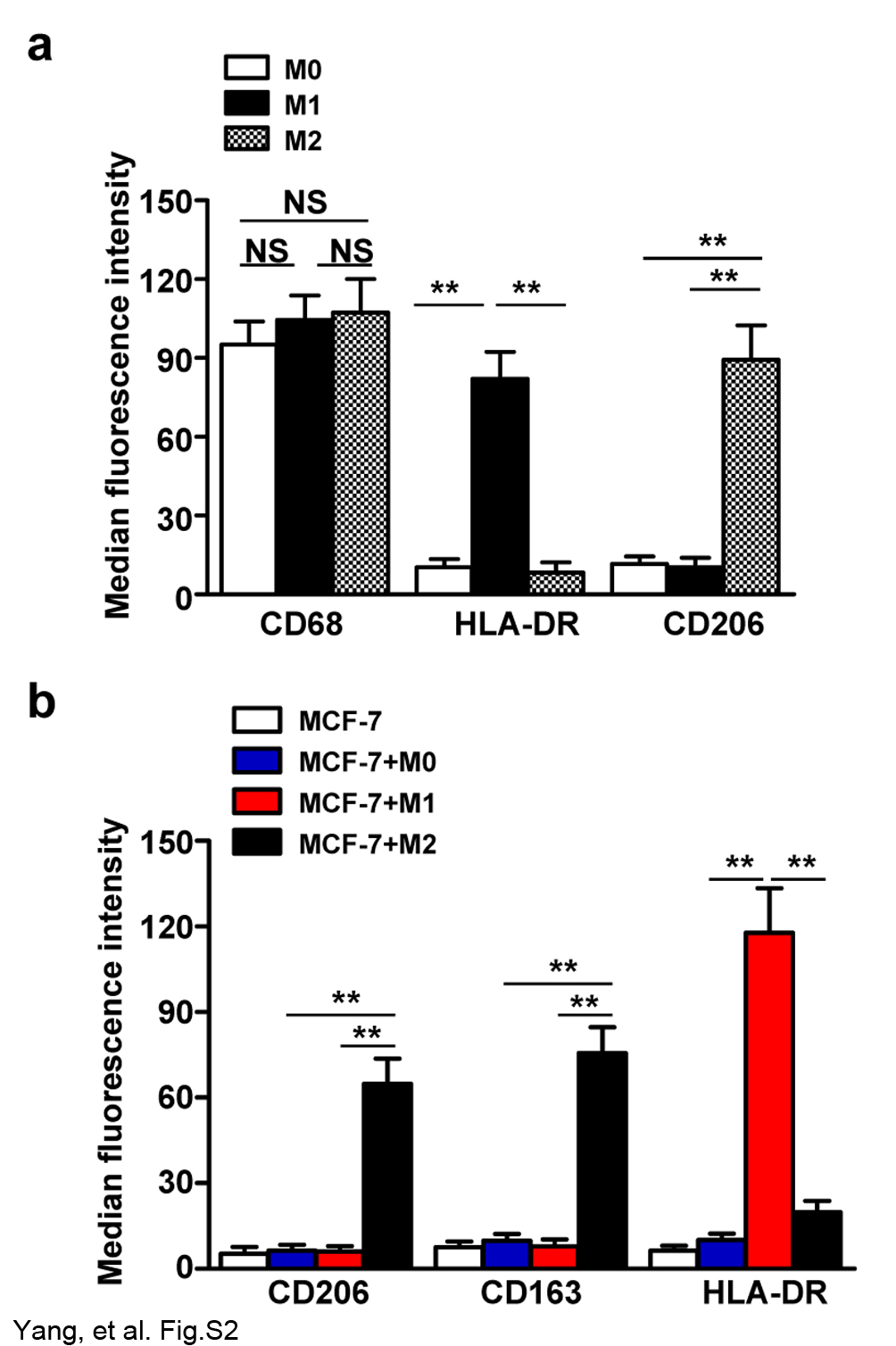

Supplement: Additional file 2: Figure S2. — Median fluorescence intensity (MFI) after staining with M1 or M2 macrophage markers and analysis by flow cytometry. The MFI served as a quantitative index of biomarker surface expression. a Quantitation of the MFI for CD68, HLA-DR, CD206 expression in M0, M1 and M2 macrophages. Data are mean ± s.e.m of three independent experiments. **, P <0.01. NS indicates not significant. b Quantitation of the MFI for CD206, CD163, HLA-DR expression in M0, M1 or M2 macrophages co-cultured with MCF-7 cells in serum-free hepatocyte maintenance medium (HMM) for 5 days, mono-cultured MCF-7 in their normal growth medium (RPMI 1640 with 10 % serum). Data are mean ± s.e.m of three independent experiments. **, P <0.01. (JPG 228 kb) [file 12885_2016_2411_MOESM2_ESM.jpg]

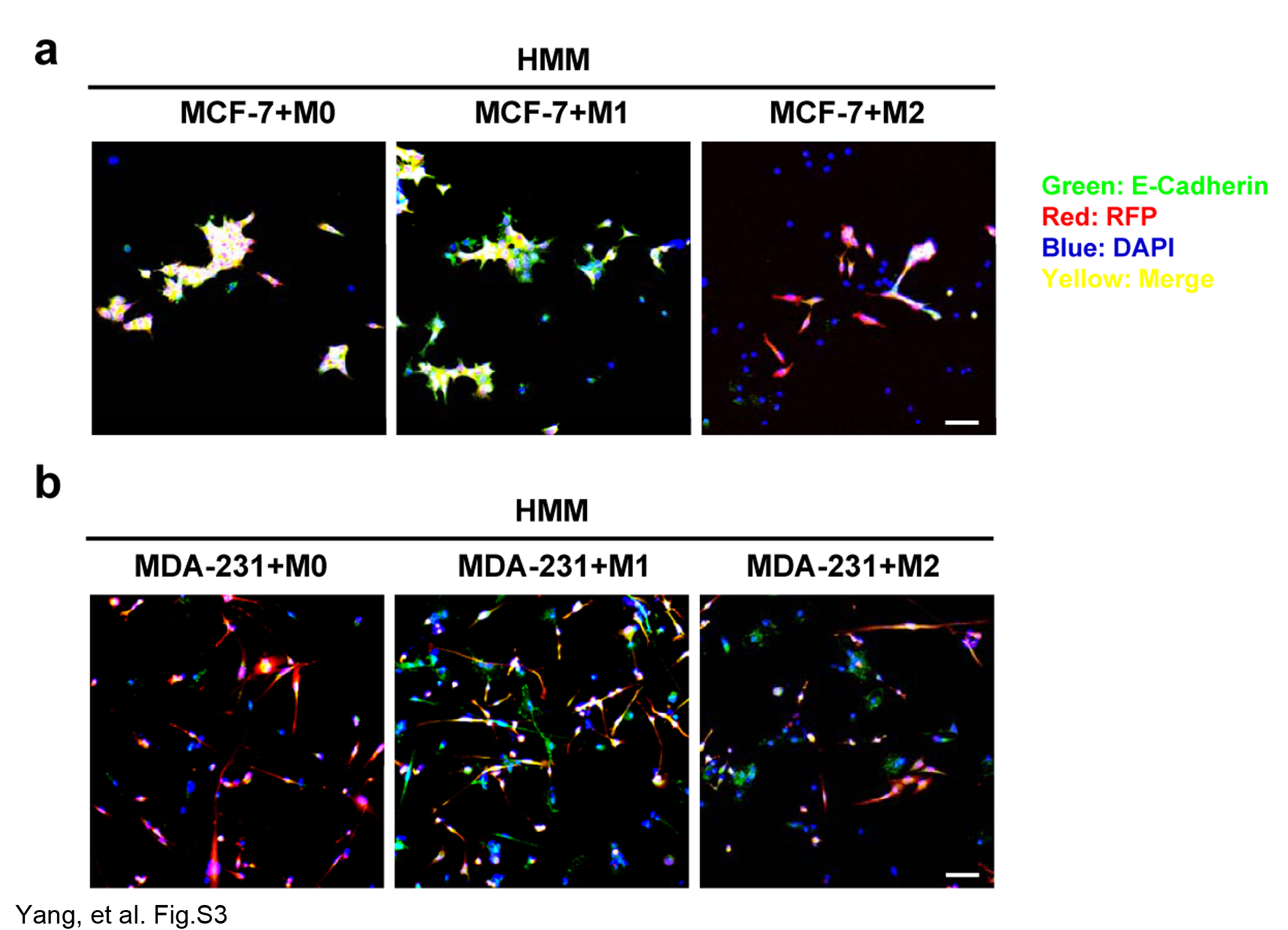

Supplement: Additional file 3: Figure S3. — E-cadherin expression of breast cancer cell co-cultured with primary macrophage cell types. a Immunofluorescence analysis of E-cadherin expression in RFP-MCF7 cells after co-culture with M0, M1 and M2 programmed human monocyte derived macrophages (MDMs) in serum-free hepatocyte maintenance medium (HMM) for 5 days. E-cadherin (green), RFP (red), DAPI (blue), Merge (yellow) (Bar = 50 μm). b Immunofluorescence analysis of E-cadherin expression in RFP-MDA-MB-231 cells by co-culture with M0, M1 and M2 programmed human monocyte derived macrophages (MDMs) in serum-free hepatocyte maintenance medium (HMM) for 5 days. E-cadherin (green), RFP (red), DAPI (blue), Merge (yellow) (Bar = 50 μm). Shown are one of two similar experiments. (JPG 588 kb) [file 12885_2016_2411_MOESM3_ESM.jpg]

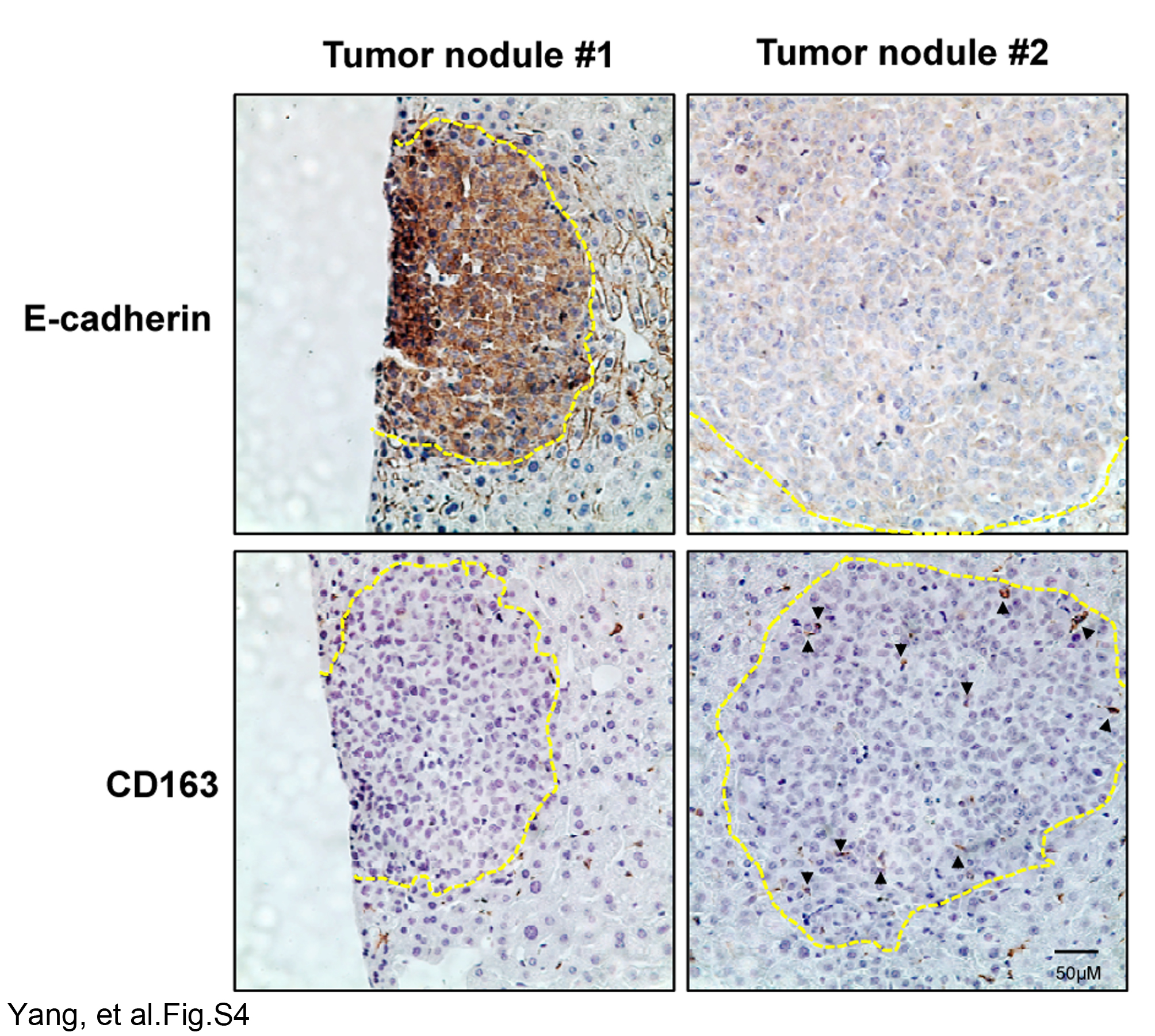

Supplement: Additional file 4: Figure S4. — Immunohistochemical analysis of E-cadherin expression or M2 macrophage marker CD163 expression in metastatic hepatic foci of mouse model of prostate cancer dissemination. Metastatic foci in the liver were obtained from NOD/SCID gamma mice at 5 weeks after intrasplenic inoculation of prostate cancer (PCa) DU145 cells. The livers were fixed in 10 % neutral buffered formalin for immunohistochemical staining. Arrows indicate CD163 positive cells. (×200 magnification and scale bar = 50 μm). Shown are representative nodules of greater than five each. (JPG 1641 kb) [file 12885_2016_2411_MOESM4_ESM.jpg]

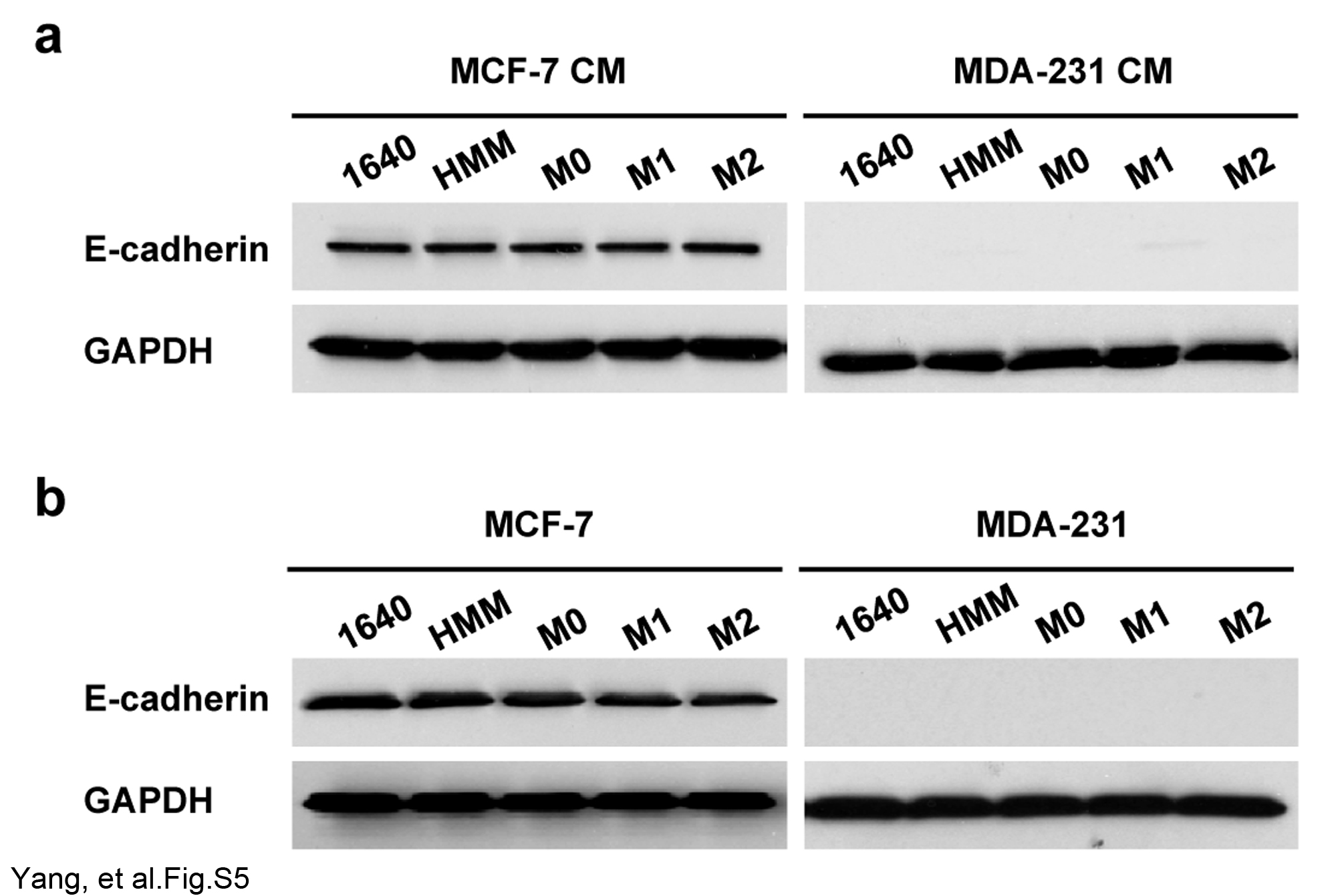

Supplement: Additional file 5: Figure S5. — Direct contact co-culture of breast cancer cells with macrophages is required to regulate epithelial-mesenchymal plasticity. a MCF-7 or MDA-231 cells were co-cultured with M0, M1 or M2 conditioned media (CM) for 5 days. b MCF-7 or MDA-231 cells were co-cultured with M0, M1 or M2 macrophages in a transwell system for 5 days. MCF-7 or MDA-231 cells were also cultured in their normal growth medium (RPMI 1640 with 10 % serum) or in serum-free HMM. Western blot analysis of E-cadherin protein expression in MCF-7 or MDA-231 cells. GAPDH was used as a loading control. (JPG 390 kb) [file 12885_2016_2411_MOESM5_ESM.jpg]
